# Supplementary material for: FastqPuri: high-performance preprocessing of RNA-seq data
Source: BMC Bioinformatics. 2019 May 3;20:226. doi: 10.1186/s12859-019-2799-0 (PMC6500068; doi:10.1186/s12859-019-2799-0)
Supplement: Supplementary file 2 — Archive of FastqPuri. Archive containing all files needed to install and run FastqPuri v1.0.6. Date stamp March 22, 2019. (GZ 47,819 kb) [file 12859_2019_2799_MOESM2_ESM.gz › FastqPuri-1.0.6/html/init__Qreport_8h_source.html]

FastqPuri: include/init\_Qreport.h Source File


FastqPuri


- include

init\_Qreport.h

Go to the documentation of this file.

1 /\*\*\*\*\*\*\*\*\*\*\*\*\*\*\*\*\*\*\*\*\*\*\*\*\*\*\*\*\*\*\*\*\*\*\*\*\*\*\*\*\*\*\*\*\*\*\*\*\*\*\*\*\*\*\*\*\*\*\*\*\*\*\*\*\*\*\*\*\*\*\*\*\*\*\*\*

2  \* Copyright (C) 2017 by Paula Perez Rubio \*

3  \* \*

4  \* This file is part of FastqPuri. \*

5  \* \*

6  \* FastqPuri is free software: you can redistribute it and/or modify \*

7  \* it under the terms of the GNU General Public License as \*

8  \* published by the Free Software Foundation, either version 3 of the \*

9  \* License, or (at your option) any later version. \*

10  \* \*

11  \* FastqPuri is distributed in the hope that it will be useful, \*

12  \* but WITHOUT ANY WARRANTY; without even the implied warranty of \*

13  \* MERCHANTABILITY or FITNESS FOR A PARTICULAR PURPOSE. See the \*

14  \* GNU General Public License for more details. \*

15  \* \*

16  \* You should have received a copy of the GNU General Public License \*

17  \* along with FastqPuri. \*

18  \* If not, see <http://www.gnu.org/licenses/>. \*

19  \*\*\*\*\*\*\*\*\*\*\*\*\*\*\*\*\*\*\*\*\*\*\*\*\*\*\*\*\*\*\*\*\*\*\*\*\*\*\*\*\*\*\*\*\*\*\*\*\*\*\*\*\*\*\*\*\*\*\*\*\*\*\*\*\*\*\*\*\*\*\*\*\*\*\*\*/

20

29 #ifndef INIT\_QREPORT\_H\_

30 #define INIT\_QREPORT\_H\_

31

32 #include "defines.h"

33

37 typedef struct \_iparam\_Qreport {

38  char \*inputfile;

39  char outputfilebin[MAX\_FILENAME];

40  char outputfilehtml[MAX\_FILENAME];

41  char outputfileinfo[MAX\_FILENAME];

42  int nQ;

43  int ntiles;

44  int minQ;

45  int read\_len;

46  int filter;

49  int one\_read\_len;

51 }Iparam\_Qreport;

52

53 void printHelpDialog\_Qreport();

54

55 void getarg\_Qreport(int argc, char \*\*argv);

56

57 #endif // endif INIT\_QREPORT\_H\_

Iparam\_Qreport

struct \_iparam\_Qreport Iparam\_Qreport

contains Qreport input parameters

\_iparam\_Qreport::minQ

int minQ

**Definition:** init\_Qreport.h:44

\_iparam\_Qreport::outputfilebin

char outputfilebin[MAX\_FILENAME]

**Definition:** init\_Qreport.h:39

\_iparam\_Qreport::outputfileinfo

char outputfileinfo[MAX\_FILENAME]

**Definition:** init\_Qreport.h:41

\_iparam\_Qreport::ntiles

int ntiles

**Definition:** init\_Qreport.h:43

\_iparam\_Qreport::inputfile

char \* inputfile

**Definition:** init\_Qreport.h:38

\_iparam\_Qreport::filter

int filter

**Definition:** init\_Qreport.h:46

\_iparam\_Qreport::nQ

int nQ

**Definition:** init\_Qreport.h:42

\_iparam\_Qreport::outputfilehtml

char outputfilehtml[MAX\_FILENAME]

**Definition:** init\_Qreport.h:40

MAX\_FILENAME

#define MAX\_FILENAME

**Definition:** defines.h:37

defines.h

Macro definitions.

printHelpDialog\_Qreport

void printHelpDialog\_Qreport()

Function that prints Qreport help dialog when called.

**Definition:** init\_Qreport.c:43

\_iparam\_Qreport::one\_read\_len

int one\_read\_len

**Definition:** init\_Qreport.h:49

\_iparam\_Qreport

contains Qreport input parameters

**Definition:** init\_Qreport.h:37

getarg\_Qreport

void getarg\_Qreport(int argc, char \*\*argv)

Reads in the arguments passed through the command line to Qreport. and stores them in the global vari...

**Definition:** init\_Qreport.c:70

\_iparam\_Qreport::read\_len

int read\_len

**Definition:** init\_Qreport.h:45


---

Generated on Mon Mar 19 2018 23:42:01 for FastqPuri by  

 1.8.14
